# Effects of Biochemical Alteration in Animal Model after Short-Term Exposure of *Jatropha curcas* (Linn) Leaf Extract

**DOI:** 10.1155/2013/798096

**Published:** 2013-05-27

**Authors:** Osamuyimen O. Igbinosa, Efosa F. Oviasogie, Etinosa O. Igbinosa, Otibhor Igene, Isoken H. Igbinosa, Omoruyi G. Idemudia

**Affiliations:** ^1^Department of Medicine, Saint Peter's University Hospital, New Brunswick, NJ, USA; ^2^Department of Microbiology, Faculty of Life Sciences, University of Benin, Benin City, Nigeria; ^3^Department of Biochemistry and Microbiology, University of Fort Hare, Private Bag X1314, Alice 5700, South Africa; ^4^College of Medicine, America University of Antigua, Antigua And Barbuda; ^5^Department of Chemistry, University of Fort Hare, Private Bag X1314, Alice 5700, South Africa

## Abstract

This study aims to evaluate potential toxic effect of *Jatropha curcas* leaves methanol extract on laboratory rats as well as determine its LD_50_. A total of 80 male Wistar rats were used as the experimental animals, 40 for LD_50_ determination and the other 40 for toxicity study. Based on the pretest that was done in order to establish a range of toxicity, 4 dosages (86.00, 58.00, 46.00, and 34.0 kg/body weight) were chosen. The rats were randomly assigned into four groups with 10 rats in each group. Rats in groups 1, 2, 3, and 4 were given 0 mg/kg, 500 mg/kg, 1000 mg/kg, and 2000 mg/kg body weight of *Jatropha curcas* extract, respectively, by oral intubation for 21 days. Thereafter, clinical signs, change in body weight, toxicity symptoms, and biochemical parameters were obtained. The LD_50_ at 95% confidence limits for rats was 46.0 mg/kg body weight (44.95–52.69 mg/kg body mass). There was no clinical and biochemical signs of toxicity when the extract was administered at 500, 1000, and 2000 mg/kg body weight, respectively (*P* > 0.05). Results obtained from this study suggest that liver, kidney, and haematological system of rats tolerated methanolic leave extract of *Jatropha curcas* at a certain concentration.

## 1. Introduction

The continued interest in the evaluation of natural products as potential chemotherapeutic agents is encouraged by the isolation of phytochemicals in the plants, which could become important drugs in modern medicine. Plants produce bioactive compounds or molecules that act as defence mechanisms against predators and at the same time may be toxic in nature [1, 2]. With the increased interest in medicinal plants, there is a need for thorough scientific investigations of these plants for efficacy and potential toxicity. 

*Jatropha curcas* (Linn) belonging to the family Euphorbiaceae is a shrub that grows 4.5 to 8 meters high. The roots, leaves, and seeds of the plant have been widely used in traditional folk medicine in many parts of West Africa, Central and South America. Previous studies have shown that the plant exhibits bioactive activities for fever, mouth infections, jaundice, and guinea worm sores [3]. Fagbenro-Beyioku et al. [4] reported antiparasitic activity of the sap and crushed leaves of *J. curcas*. The water extract of the branches also strongly inhibited HIV-induced cytopathic effects with low cytotoxicity [5]. Mujumdar et al. [6] also reported that the crude methanol extract from the root of *J. curcas* exhibited antidiarrheal activity in mice through inhibition of prostaglandin biosynthesis and reduction of osmotic pressure. Our biological study on *J. curcas* reported relevant antimicrobial efficacy and antioxidant activities [7, 8]. Balaji et al. [9] reported that methanol extract of *J. curcas* could protect liver against the aflatoxin B1-induced oxidative damage in rats. Despite all beneficial effects of *J. curcas*, some studies have also demonstrated that *J. curcas *exhibited toxicity especially in higher animals. For example, methanol, petroleum ether, and dichloromethane extracts of *J. curcas* fruit caused fetal resorption indicating pregnancy terminating effect in rats [10]. Methanol fraction from *J. curcas* oil induced tumor promotion upon topical initiation by Makkar et al. [11], dimethylbenz(a)anthracene (DMBA) in mice, with 36% of the animals having skin tumors in 30 weeks [12]. Raw or defatted seeds when administered to fish, chicks, pigs, goats, mice, and rats were associated with toxic symptoms before death [13, 14]. Different aqueous extracts also exhibited different toxic symptoms depending on dose, mode of administration, and sensitivity of the animals that were tested [15, 16]. 

 In recent times, concerns have been raised over the lack of quality control and scientific facts for the efficacy and safety of medical plants [17, 18]. Cautions have been raised regarding the potential adverse effects of herbal remedies including hepatotoxicity and nephrotoxicity [19, 20], even as it is known that medicinal plants typically contain several different pharmacologically active compounds that may act individually, additively, or in synergy to improve health [8, 21, 22]. It has been reported that 80% of the population in the developing world still rely on traditional medicine for primary health care needs. In spite of the diverse uses of plants in folk medicine, there seems to be dearth of information on the possible toxicity of this plant. Therefore, this study evaluates the toxicity risk of the methanol extract of the plant leaves of *J. curcas* using animal model.

## 2. Results and Discussion

### 2.1. Clinical Signs and Mortality

 Death of rats administered with *J. curcas* extract occurred at a dose-dependent manner with starting dose of 34 mg/kg (Table 1). At the highest dose of 86.0 mg/kg, majority of rats were easily affrighted and stayed crouched together and tend not to eat much. Before rats died, they exhibited signs of depression, closing of eyes, languishment, loss of body mass, and black excreta. Rats began to die on day 2 after administering *J. curcas* extract, continued with a majority of deaths occurring within 7 days. There was no death recorded between observation days, 12 and 21 days. 

Table 2 shows the effect of *J. curcas* extract on weight gain, food intake, and fecal output. The result obtained indicates that weight gain, food intake, and fecal output of rat treated with 500, 1000, and 2000 mg/kg body weight of extract were not significantly different (*P* > 0.05). The effects of oral administration of the leaf extract of *J. curcas* at the doses investigated on RBCs and its functional indices in laboratory rats (Wistar) for 21 days are shown in Table 3. The extract did not significantly alter the level of Hg, RBC, PCV, MCHC, and LUC. The administration of the extract effectively reduced the level of WBC and the differentials including basophils, monocytes and platelets throughout the study period (Table 3). 

The plant extract showed varied effects on the kidney and its functional indices (Table 3). The levels of sodium, potassium, calcium, urea, and creatinine were not significantly affected when compared with the control animal model. In contrast, however, the level of chlorine ion was decreased as doses dependent factor. The extract did not significantly alter the level of albumin and total bilirubin which is the vital assay in assessing liver damage (Table 3). There was no significant difference in the parameters measured in rats administered with 500, 1000, and 2000 mg/kg body weight of *J. curcas* extract. These parameters include alanine aminotransferases (ALT), aspartate aminotransferases (AST), alkaline phosphatase (ALP), gamma glutamyl transferase (GGT), and blood urea nitrogen (BUN) (Table 3).

### 2.2. Discussion

*J. curcas* has been a multipurpose perennial plant which has lots of industrial and a long history of various medicinal applications. Antimicrobial and antioxidant activities of *J. curcas* were scientifically established [7, 8]. Since several reports have highlighted toxicity of this plant, this study ascertains safe dose of methanolic leave extract of *J. curcas* using animal model.

 There are many methods used in calculating LD_50_ such as the graphical method of Miller and Tainter, arithmetical method of Karber, and statistical approach which include up-and-down procedure, fixed dose procedure, acute toxic class method, Bliss method, and sequential grouping method. Bliss [23] developed the idea of transforming the sigmoid dose-response curve to a straight line. In 1952, Finney popularized Bliss' idea in a book called *Probit Analysis *[24]. Bliss method being classical is still the preferred statistical method. Li et al. [25] in understanding dose-response relationship the procedure was used in our study.

 LD_50_ study indicated that *J. curcas* leave extract is toxic to rats at a high dose, and rats develop severe pathological symptoms. The obtained lethal dose as shown in this study may not predict the human lethal dose of a drug or acute poisoning overdose. However, it was used to provide a guideline for selecting doses for subacute dosage of more clinical relevance. Feeding studies on *J. curcas* showed severe clinical and pathological symptoms in a dose-dependent manner [26, 27]; symptoms observed include transient loss of body mass and mild to severe macroscopic and microscopic changes in the kidney, lungs, heart, liver, and spleen. In the present study, the observed dark excreta may be suggestive of gastrointestinal hemorrhage.

 The treatment of animals at doses 500, 1000, and 2000 mg/kg for 21 days show selective toxic effect on some biochemical and hematological parameters. These parameters are used to determine the possible alterations in the level of biomolecules such as enzymes, metabolic product, normal functioning and histomorphology of the organs. There were no noticeable hemolytic changes in the plasma of the extract treated rats on RBC, Hg, PCV, MCHC, and LUC. These indices are well known to determine the hemolytic damage on RBCs. The absence of changes on these functional properties suggests that the extract does not possess toxic substances that can cause anemic condition in rats. A decreased hemoglobin and hematocrit levels, indicative of anemia, is associated with hemolysis from antigen-antibody response. Rise in white blood cell (WBC) is generally considered to be a marker of stress and a defence mechanism triggered by immune system. 

 In this study, there was no significant difference in WBC and hemoglobin in all observed groups. The blood creatinine or urea nitrogen levels are indicative of renal function [28]. Awasthy et al. [29] reported significant increase in creatinine of rats when basal feed was supplemented with 25% and 50% Jatropha seed protein. However, administering *J. curcas *at doses far below lD_50_ (500, 1000, and 2000 mg/kg body) in this study was not associated with significant change in weight, liver chemistry, and hematologic profile when compared with control. 

 Elevated serum transaminase activity is highly suggestive of hepatic impairment in animals [30]. Serum transaminases was reported to be significantly elevated in goats fed with *J. curcas *seeds at 0.25 to 10 g/kg/day up to 21 days; 23 in calves orally administered *J. curcas* seeds in suspension at 0.25, 1.0, and 2.5 g/kg within 14 days [31]. Also desert sheeps were fed with the seeds at 0.5 and 1.0 g/kg/day [32]. The common denominator in the above toxicity studies is that they were all administered seeds. Our current study on *J. curcas* leaves did not show any significant increase in serum transaminase. This observation may be due to lower concentration phorbol esters in *J. curcas* leaves, exposure time, dose, and extraction type (methanolic extracts). Several compounds have been isolated from *J. curcas* seeds, it is reasonable to extrapolate that these compounds are present at lower concentration in the leaves as well. The compounds include saponins, lectins (curcin), phytates, protease inhibitors, curcalonic acid, and phorbol esters, but studies that isolate phorbol esters in toxic and non toxic strains have determined that phorbol esters as compounds of concern [28]. Phorbol esters are present in leaves, stems, flowers, and roots of *J. curcas* [11]. 

## 3. Experimental Section 

### 3.1. Collection and Identification of Plant Materials

 Fresh leaves of *J. curcas* were collected from a local farm in Benin City, Edo State, Nigeria, in the month of June 2010 and were identified by the Botany Department of Ambrose Alli University, Ekpoma, Nigeria. 

### 3.2. Extraction of Plant Materials

 Powdered plant materials (100 g of each) were extracted with 1000 mL methanol in a Soxhlet apparatus for 8 hours. The obtained methanolic extracts were filtered and evaporated by using a rotary evaporator and freeze dryer to give the crude dried extract.

### 3.3. Experimental Animals

 Total of 80 male Wistar (8 weeks old) rats were used as the experimental animals; 40 for LD_50_ determination and the other 40 for toxicity study. The animals were maintained in a room with controlled temperature (35 ± 2°C) for 12 h light/12 h dark cycle with food (standard pellet diet) and sterile water provided *ad librium*. The animals were habituated to the experimental room for at least 24 h before the experiments. After acclimatization, the mice were weighed and numbered. The experiment was conducted in a barrier system with an experimental facility. Animal studies were in compliance to the ethical procedure for the care and use of laboratory animals that corresponds with NIH guidelines.

### 3.4. Assay Kits

 The assay kits for total protein, creatinine, urea, calcium, sodium, potassium, chloride, albumin, bilirubin, alkaline phosphatase (ALP), gamma glutamyltransferase (GGT), alanine aminotransferases (ALT), and aspartate aminotransferases (AST) were obtained from Bayer's Diagnostics, Baroda, India. 

### 3.5. Determination of LD_50_ of the Extract

 Rats weighing 102 ± 4.8 g were used for the determination of LD_50_ of the extract. Based on dose levels that decrease in geometrical progression, the regression equation between the probits of mortalities (*Y*) and the log of doses (*D*) was also derived: *Y* (probit) = −9.67 + 10.21 log (*D*). Four (4) doses (86.00, 58.00, 46.00, and 34.0 kg/body mass) were ultimately required for establishing the LD_50_. Calculation of LD_50_, was based on 95% confidence limits [33]. The Bliss was calculated by using the NDST Software Version 8.0 [34].

### 3.6. Toxicity Study Design

 A pretest was conducted to observe the range of toxicity in others to establish dose range for LD_50_ determination. Three dose levels (6, 12, and 18 mg/kg body mass) of methanoic leave extract of *J. curcas* were used for the pretesting. Based on the pretest results, 4 dosages (86.00, 58.00, 46.00, and 34.0 kg/body mass) were established with each group comprised of 10 rats using random block design. 

 The rats were randomly assigned into four groups with 10 rats in each group. Rats in groups 2, 3, and 4 were given 500, 1000, and 2000 mg/kg body weight, respectively, orally by intubation. Control rats (group 1) were administered the same volume of deionized water. The doses administered were below the LD_50_ of the extract which was found to be 46.0 g/kg body weight of rat.

 The rats were fed daily for two weeks. At the end of study period after fasting for 3 hours, each rat was anaesthetized in chloroform-saturated chamber. Under anesthesia, the abdominal and thoracic region of each rat was opened to assess the heart. The blood samples were collected through cardiac puncture in properly heparinized vials. The tube was swirled and placed on ice. Plasma was obtained from blood by centrifugation at 3000 rpm for 5 min. The different biochemical parameters were analyzed from blood plasma by using the diagnostic kits (Bayer's Diagnostics, Baroda, India) with the help of a semiautomated analyzer (RA-50 chemistry). Clinical signs, change in body mass, and toxicity symptoms were observed daily for 21 days.

### 3.7. Statistical Analysis

 Experimental data were expressed as mean of six replicates and subjected to one way analysis of variance (ANOVA). Means were separated by Duncan multiple range test using the Statistical Analysis System (SAS version 8, SAS Institute, Cary, NC, USA). Values were considered statistically significant level at *P* < 0.05.

## 4. Conclusions 

 In conclusion, although direct extrapolation of results from animal models cannot be applied to humans, results obtained from this study suggest that the liver, kidney, and hematological system of rats tolerated leave extract of *J. curcas* at a certain concentration. However, histopathology study is desirable to confirm these findings. One issue that remains unsolved is the efficacy of this plant under investigation at nontoxic doses.

## Figures and Tables

**Table 1:** Determination of the LD_50_ value.

Dose (mg/kg)	Total dead	Days after administration												
		0	1	2	3	4	5	6	7	8	9	10	11	12–21
86.0	9	0	0	2	0	1	1	1	2	1	0	0	1	0
58.0	7	0	0	0	1	1	2	2	0	0	1	0	0	0
46.0	4	0	0	1	0	1	0	0	1	0	0	0	1	0
34.0	2	0	0	0	0	0	1	0	0	0	0	1	0	0
Control	0	0	0	0	0	0	0	0	0	0	0	0	0	0

**Table 2:** Weight gain, food consumption, and fecal output of rats administered *J. curcas* extract (*n* = 6, mean ± SD).

Parameter	Dose of extract (mg/kg body weight)			
	Control	500	1000	2000
Weight gain (g/day/rat)	1.52 ± 0.23^a^	1.64 ± 0.45^b^	1.57 ± 0.65^b^	1.2 ± 0.59^d^
Food intake (g/day/rat)	21.34 ± 1.32^a^	24.93 ± 0.32^b^	25.01 ± 1.24^d^	23.82 ± 1.51^e^
Dry fecal output (g/day/rat)	1.34 ± 0.09^a^	1.29 ± 0.05^c^	1.41 ± 0.06^c^	2.01 ± 0.03^e^

Means with the same letter are not significantly different (*P* < 0.05).

**Table 3:** Effects of methanolic leaf extract of *J. curcas *on plasma biochemical parameters of laboratory rats (*n* = 6, mean ± SD).

Parameter	Doses of extract (mg/kg body weight)			
	Control	500	1000	2000
ALT (U/L)	21.03 ± 0.47^a^	19.93 ± 0.83^ab^	22.03 ± 0.93^ab^	25.07 ± 1.85^a^
AST (U/L)	19.05 ± 1.03^a^	24.05 ± 0.93^a^	25.47 ± 0.46^ab^	24.93 ± 1.09^a^
ALP (U/L)	43.03 ± 0.84^a^	48.03 ± 1.03^a^	45.04 ± 1.39^a^	51.09 ± 3.04^a^
GGT (U/L)	28.72 ± 3.29^a^	29.54 ± 4.12^ab^	30.60 ± 2.46^a^	33.62 ± 3.01^ab^
Albumin (mmol/L)	19.50 ± 0.85^a^	19.12 ± 0.15^a^	18.56 ± 1.74^a^	17.31 ± 2.10^a^
Total bilirubin (*μ*mol/L)	12.53 ± 0.71^a^	11.38 ± 0.21^a^	11.01 ± 2.08^a^	9.50 ± 0.53^a^
Total protein (g/L)	6.07 ± 0.52^a^	7.99 ± 0.62^b^	5.80 ± 0.32^b^	5.94 ± 0.46^a^
BUN (mg/dL)	8.03 ± 2.01^a^	10.90 ± 1.84^ab^	9.91 ± 0.73^a^	11.05 ± 1.89^b^
Glucose (mmol/L)	5.59 ± 0.01^a^	5.78 ± 0.01^a^	5.71 ± 0.02^a^	5.40 ± 0.20^a^
Potassium (mmol/L)	6.95 ± 1.13^a^	5. 52 ± 0.18^a^	5.94 ± 0.15^a^	5.96 ± 0.25^a^
Sodium (mmol/L)	145.33 ± 2.51^a^	146.52 ± 3.17^a^	143.76 ± 0.68^a^	141.98 ± 0.82^a^
Chloride (mmol/L)	125.5 ± 3.52^a^	110.45 ± 2.53^ab^	98.51 ± 1.15^a^	95.78 ± 1.58^a^
Calcium (mmol/L)	5.54 ± 0.12^a^	4.85 ± 0.01^a^	5.01 ± 0.05^b^	5.15 ± 0.04^a^
Urea (mmol/L)	6.08 ± 0.21^a^	7.89 ± 1.50^a^	7.45 ± 0.58^a^	6.50 ± 0.15^a^
Creatinine (mmol/L)	0.56 ± 0.23^ab^	0.73 ± 0.23^b^	0.48 ± 0.35^a^	0.66 ± 0.34^b^
WBC count (×10^9^/L)	16.20 ± 2.5^a^	7.53 ± 1.60^b^	9.02 ± 1.5^b^	8.9 ± 2.4^b^
Platelets (×10^9^/L)	789.45 ± 5.84^a^	734.01 ± 2.52^c^	650.53 ± 7.89^c^	645.32 ± 3.50^b^
Basophils (%)	0.85 ± 0.51^a^	0.55 ± 0.01^a^	0.53 ± 0.21^a^	0.50 ± 0.35^a^
Monocytes (%)	27.56 ± 3.21^a^	15.65 ± 4.50^c^	12.89 ± 5.01^c^	18.98 ± 2.51^b^
Hg (g/L)	12.80 ±1.6^ab^	10.04 ± 2.04^a^	9.03 ± 2.09^ac^	8.9 ± 1.76^ab^
RBC (×10^9^/L)	8.75 ± 0.05^a^	9.84 ± 0.51^a^	8.70 ± 0.53^a^	8.45 ± 0.15^a^
PCV (L/L)	0.50 ± 0.03^a^	0.52 ± 0.2^bc^	0.48 ± 0.02^ab^	0.47 ± 0.04^ac^
MCHC (g/dL)	29.50 ± 1.52^a^	32.55 ± 0.52^a^	31.81 ± 1.20^a^	31.50 ± 0.51^a^
LUC (%)	8.95 ± 1.05^a^	10.25 ± 0.78^a^	9.85 ± 1.07^a^	8.45 ± 0.36^a^

Means with the same letter are not significantly different (*P* < 0.05).

Legend: alanine transaminase (ALT); aspartate transaminase (AST); alkaline phosphatase (ALP); gamma glutamyl transferase (GGT); blood urea nitrogen (BUN); haemoglobin (Hg); pack cell volume (PCV); white blood cell (WBC); red blood cell (RBC); mean corpuscular haemoglobin concentration (MCHC); large unstained cell (LUC).
